# Comparison of overtly and covertly observed hand antisepsis adherence in the intensive care units of a referral hospital in Iran

**DOI:** 10.3205/dgkh000593

**Published:** 2025-11-14

**Authors:** Farid Zand, Anahita Sanaei, Naeimehossadat Asmarian, Fatemeh Zarei, Azita Tabatabai, Rahele Zandi, Solmaz Salami, Kowsar Shoja

**Affiliations:** 1 Anesthesiology and Critical Care Research Center, Shiraz University of Medical Sciences, Shiraz, Iran; 2 Clinical Microbiology Research Center, Shiraz University of Medical Sciences, Shiraz, Iran; 3 Infection Control Team, Namazi Hospital, Shiraz, Iran

**Keywords:** hand antisepsis, overt observation, covert observation, adherence

## Abstract

**Background and objective:**

Hand antisepsis one of the most important strategies for preventing transmission of drug-resistant microorganisms and healthcare-associated infections. Overt observation of hand antisepsis in healthcare settings is currently considered the gold standard for monitoring its adherence rate. However, a major limitation of this method is the “Hawthorne effect”, wherein health staff who know they are being assessed may alter their behavior. In this survey, we aimed to compare the overt and covert methods of observation of hand hygiene.

**Method:**

This cross-sectional descriptive-analytic study was conducted over six months in the intensive care units (ICUs) of Namazi Teaching Hospital in Shiraz. The study population comprised ICU staff, including nurses, physicians (specialists and residents), and other healthcare workers (HCWs) in direct contact with patients. Convenience sampling was used. Hand antisepsis adherence was assessed through both overt (open) and covert (hidden) observations across different work shifts. Data was collected using the “WHO Hand Hygiene Observation Checklist” and analyzed in SPSS 18 using descriptive statistics and the chi-squared test.

**Results:**

The findings revealed that in a total of 9 ICUs (including 4 surgical, 2 medical, and 3 general ICUs) with over 90 active beds, 776 opportunities were observed through overt observation and 1,780 opportunities through covert observation. Overall, hand antisepsis adherence was 58.4% in overt observation and 60.3% in covert observation (p=0.352, RR=0.96). A significant difference was found in adherence rates among certain professional groups and specific hand antisepsis opportunities.

**Discussion and conclusion:**

No significant difference was found in hand antisepsis adherence between overt and covert observation methods, suggesting that both approaches are suitable to assess the in health care centers. It is essential to institutionalize hand antisepsis as an occupational culture in healthcare settings. Raising awareness among healthcare teams about the severe consequences of hospital-acquired infections can provide the necessary motivation for consistent and proper hand antisepsis practices.

## Zusammenfassung

**Hintergrund und Zielsetzung:**

Die Händeantiseptik ist eine der wichtigsten Strategien zur Verhinderung der Übertragung multiresistenter Mikroorganismen und nosokomialer Infektionen. Die offene Beobachtung der Händeantiseptik in Gesundheitseinrichtungen gilt derzeit als Goldstandard für die Überwachung ihrer Einhaltung. Eine wesentliche Einschränkung dieser Methode ist jedoch der „Hawthorne-Effekt“, bei dem Gesundheitspersonal, das weiß, dass es bewertet wird, sein Verhalten ändern kann. In dieser Umfrage wollten wir die offenen und verdeckten Methoden zur Beobachtung der Händehygiene vergleichen.

**Methode:**

Die deskriptiv-analytische Querschnittsstudie wurde über einen Zeitraum von sechs Monaten auf den Intensivstationen (ICUs) des Namazi Teaching Hospital in Shiraz durchgeführt. Die Studienpopulation bestand aus ICU-Mitarbeitern, darunter Krankenschwestern, Ärzte (Fach- und Assistenzärzte) und andere Gesundheitsfachkräfte, die in direktem Kontakt mit Patienten standen. Es wurde ein Convenience-Sampling verwendet. Die Einhaltung der Händeantiseptik wurde durch offene und verdeckte Beobachtungen während verschiedener Arbeitsschichten bewertet. Die Daten wurden mithilfe der „WHO-Checkliste zur Beobachtung der Händehygiene” erhoben und in SPSS 18 unter Verwendung deskriptiver Statistiken und des Chi-Quadrat-Tests analysiert.

**Ergebnisse:**

Insgesamt wurden auf 9 Intensivstationen (4 chirurgische, 2 medizinische und 3 allgemeine ICUs) mit über 90 aktiven Betten 776 Gelegenheiten durch offene Beobachtung und 1.780 Gelegenheiten durch verdeckte Beobachtung beobachtet. Insgesamt betrug die Adhärenz der Händeantiseptik bei offener Beobachtung 58,4%, bei verdeckter Beobachtung 60,3% (p=0,352, RR=0,96). Es wurde jedoch ein signifikanter Unterschied in den Adhärenz-Raten zwischen bestimmten Berufsgruppen und spezifischen Gelegenheiten der Händeantiseptik festgestellt.

**Diskussion und Schlussfolgerung:**

Es wurde kein signifikanter Unterschied zwischen der offenen und verdeckten Beobachtungsmethode hinsichtlich der Einhaltung der Händeantiseptik festgestellt, was darauf hindeutet, dass beide Ansätze zur Bewertung der Einhaltung in Gesundheitseinrichtungen geeignet sind. Es ist unerlässlich, die Händeantiseptik als Arbeitskultur in Gesundheitseinrichtungen zu institutionalisieren. Die Sensibilisierung der Gesundheitsteams für die schwerwiegenden Folgen von nosokomialen Infektionen kann die notwendige Motivation für eine konsequente und ordnungsgemäße Händeantiseptik liefern.

## Introduction

In 2004, the World Health Organization (WHO) started the global Hand Hygiene Initiative to reduce healthcare-associated infections (HAIs) and improve patient safety [1], [2]. Hand antisepsis is one of the most crucial strategies to prevent HAIs and transmission of drug-resistant pathogens, both of which contribute to significant morbidity and mortality among hospitalized patients [3], [4].

Around 7.5% of hospitalized patients in developed countries and 10% in developing countries are affected by HAIs. 20–40% of these infections are a direct consequence of transmission of pathogens via the contaminated hands of healthcare staffs [5], [6].

Despite being simple and cost-effective, hand antisepsis adherence remains inadequate in many healthcare settings, both in quality and quantity. Some methods can assess hand antisepsis adherence, including direct observation, self-reporting and indirect monitoring (e.g., measuring hand-rub usage per unit or individual) [7].

Direct observation – although it is the gold standard – has a major limitation: the Hawthorne effect, where HCWs alter their hand antisepsis behavior when they know they are being observed, influencing the results of the assessment. While this often overestimates adherence, only a few studies have evaluated the Hawthorne effect using explicit observation. To address this, covert (unannounced) observation has been proposed to overcome the bias of the Hawthorne effect [4], [8]. Accordingto a WHO report, global hand antisepsis adherence rate is around 80%. The rate at our university hospital is about 62%, in spite of implementing WHO’s strategies. Factors like training quality and quantity, cultural differences, workload, ethics, and limited supervision and monitoring (only during morning shifts by infection control supervisors) may explain this gap.

Considering the higher cost and labor involved in covert observation compared to overt assessments, and the necessity of comparing it with overt method, this study was designed and conducted in adult ICUs. The research also compares hand antisepsis adherence rates across different hospital shifts, providing insights to improve the situation.

## Study design and method

A prospective descriptive-analytic cross-sectional study was conducted over a six-month period in the adult intensive care units (ICUs) of Namazi University Hospital in Shiraz, Iran. Namazi hospital includes nine adult ICUs with a total of 90 active beds. The study population consisted of ICU healthcare personnel, including nurses, physicians (specialists and residents), and other clinical and support staff from laboratory and radiology units who were directly involved in the care of ICU patients. A convenience sampling method was used.

This research was conducted in accordance with the ethical standards of the institutional and/or national research committee and received ethical approval from the Ethics Committee of [Shiraz University of Medical Sciences Ethics Committee], approval code [IR.SUMS.REC.1402.344].

Hand hygiene adherence was assessed using both overt and covert observation methods. Data collection was conducted using the World Health Organization (WHO) standardized hand hygiene observation checklist, which includes three main sections:

1. The five moments for hand antisepsis: Before touching a patient, before aseptic procedures, after exposure to bodily fluids, after touching a patient, after touching the patient’s surroundings [9].
2. Demographic and professional information, including gender and professional role (e.g., nurse, physician, auxiliary/support staff, and other medical staff such as physiotherapists, radiology or lab technicians).
3. Hand antisepsis technique assessment: This section assessed whether the hand antisepsis procedure was performed adequately or inadequately. A hand rub action was recorded as "correctly performed" if two conditions were met:

- The recommended duration was followed (20–30 seconds for alcohol-based hand rub). Previously, washing hands for 40–60 seconds with soap and water was also recommened. However, it has since been shown that washing hands with soap and water does not achieve the same effectiveness as alcohol based hand rubs (ABHR), is not an alternative for hand antisepsis and has therefore been abandoned internationally.
- All recommended hand areas were covered (palms, backs of hands, between fingers, four fingers, and thumbs). If either of these criteria was not fulfilled, the action was recorded as "not correctly performed."

For overt observations, infection control supervisors or designated infection control personnel conducted the assessments. Based on a pre-scheduled Gantt chart, each ICU was selected and the observer introduced themselves to the staff before conducting 20–30 minutes of hand antisepsis monitoring. The results were then entered into the RASTAK system, an integrated quality improvement data platform used at Namazi hospital.

In the covert observation method, anonymous nursing staff of each unit conducted the survey. They were adequately trained in how to observe and complete the forms, and recorded observations inconspicuously during their regular shifts while performing their duties without identifying themselves as observers. After data collection, the completed forms were submitted to the Anesthesiology and Critical Care Research Center where a trained staff member entered the data into the INICC system (International Nosocomial Infection Control Consortium). This is a global platform for monitoring and controlling HAIs, and the collected data were uploaded to its online system.

Data were analyzed using SPSS version 18, employing descriptive statistics and the Chi-squared test to evaluate associations.

## Results

A total of 2,556 opportunities were observed: 776 opportunities during 142 observation sessions using overt observation and a total of 1,780 opportunities during 143 sessions using covert observation (Table 1 (Tab. 1) ). For the two observation methods, the majority of those observed were nurses (73.9% and 48.9%, respectively). The highest hand antisepsis adherence in overt observation was related to the surgical ICU, and the highest in covert observation was related to the general ICU (45% and 32.1%, respectively). In overt observation, all observations were during the day and yielded 58.4%; covert observation yielded 60.2% during the day and 60.4% during the night. In total, 80% of hand antisepsis, overtly observed, was performed by females, and in covert observation, this rate was 55.8%. In overt observation, out of 776 opportunities, 58.4% performed hand antisepsis. In covert observations, of 1074 opportunities, the rate of hand antisepsis carried out was 60.3%.

Overall, in overt observation, hand antisepsis adherence before patient contact was 44.3%, followed by 65.8% after patient contact, 22% after contact with the patient's surroundings, 66.5% before performing aseptic tasks, and 84.6% after exposure to bodily fluids. In covert observation, hand antisepsis adherence before patient contact was 41.5%, followed by 73.9% after patient contact, 46.9% after contact with the patient's surroundings, 66.7% before performing aseptic tasks, and 85.5% after exposure to bodily fluids.

**Figure 1:**
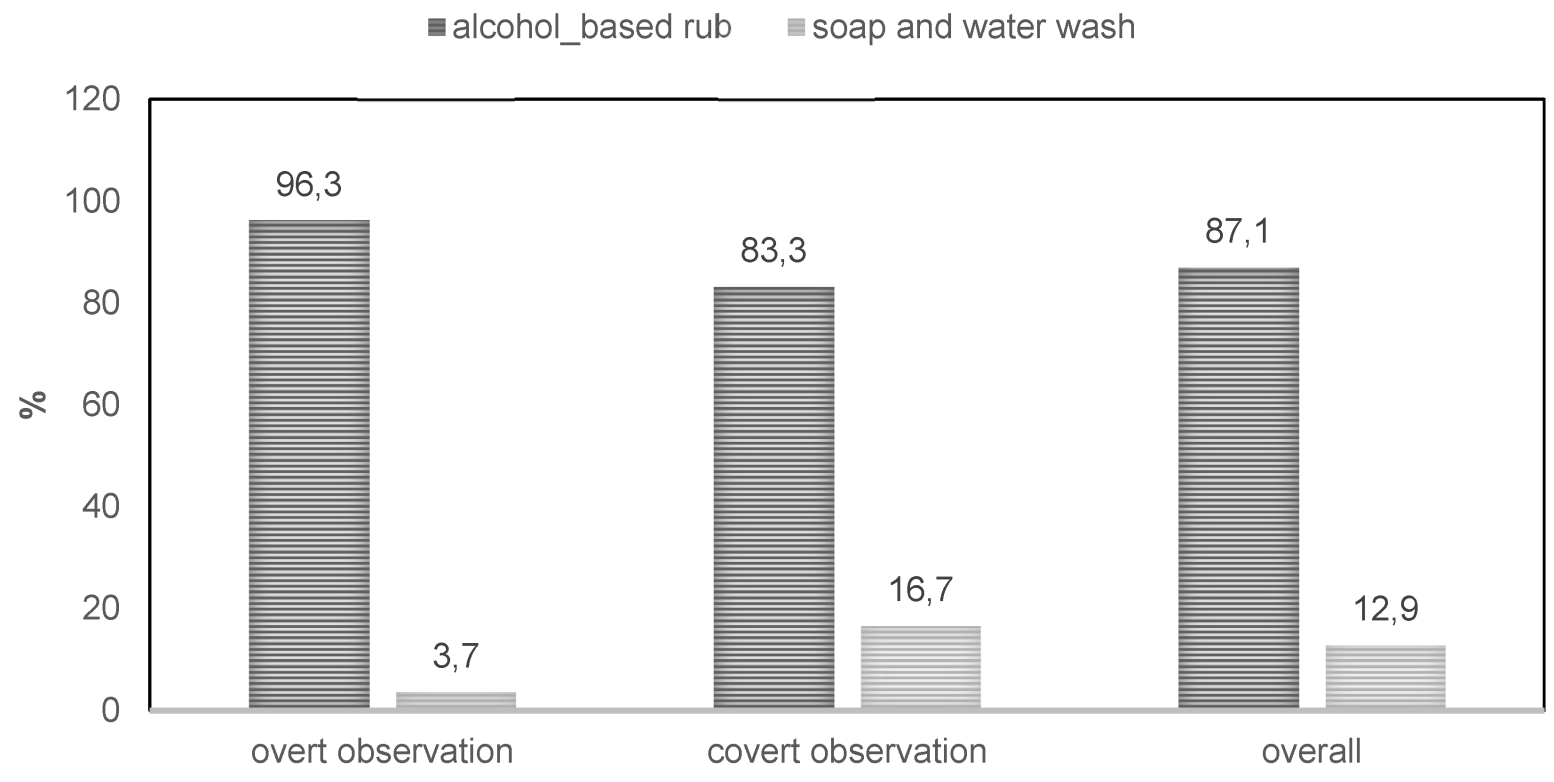
Type of hand washing based on observation type

Comparing the type of hand washing between the two observation methods, the overtly observed use of ABHR was 96%, compared to 83% in covert observation. In general, the use of ABHR was much higher than soap and water (87.1% vs. 12.9%; Figure 1 (Fig. 1)).

As shown in Table 1 (Tab. 1), after classification by occupational category, the rate of hand antisepsis adherence in the physician group under covert observation was slightly higher than when overtly observed (p<0.0001). In the overt observation method, there was a statistically significant relationship between occupational categories (p=0.0001), with nurses exhibiting the highest adherence rate. In the covert observation method, there was also a statistically significant relationship (p=0.0034), i.e., physicians had the highest adherence rate. Hand antisepsis adherence among nurses overtly observed was 62.4% compared with 64.1% measured using covert observation (RR 0.97; p=0.463). In overt observation, other healthcare providers had the highest hand antisepsis adherence followed by physicians, but in covert observation, physicians demonstrated higher rates of compliance than other healthcare providers (p=0.13); (p<0.0001). After stratification by ICUs, hand antisepsis adherence in a surgical ICU was slightly higher in covert observation than in overt observation (p<0.0001). In overt observation, there was no statistically significant relationship between hospital wards (p=0.8080), with the lowest hand antisepsis adherence in surgical ICUs and the highest adherence in internal ICUs. In covert observation, a statistically significant association existed (p=0.0045), with the lowest rate of hand antisepsis adherence in the internal ICU and the highest rate in the surgical ICU. The difference between the two observation methods varied more across the different hospital ICUs than across different job categories. For example, the hazard ratio ranged from 1.14 in the internal ICU to 0.79 in the surgical ICU.

There was no statistically significant difference in hand antisepsis adherence between covert and overt observations, but there was a tendency and bias towards covert observation (Table 2 (Tab. 2)). For example, hand antisepsis adherence for before-patient contact was 44.3% using overt observation and 41.5% using covert observation (RR 1.06). The difference between the two observation methods was that in overt observation, the highest rate of hand antisepsis adherence was after exposure to bodily fluids (84.6%) and the lowest rate was after contact with the patient's environment (22%). In covert observation, the highest rate of hand antisepsis adherence was after exposure to bodily fluids (85.5%) and the lowest rate was before contact with the patient (41.5%). In addition, there was a large variation in hand antisepsis adherence across hand antisepsis situations in both observation methods. For example, hand antisepsis was highest after contact with bodily fluids and lowest before patient contact, measured using both observations. Broadly similar findings were observed after classifying hand antisepsis situations by occupational category (Table 2 (Tab. 2)).

## Discussion

This study reveals that hand antisepsis adherence in the surveyed ICUs remains suboptimal, particularly before patient contact and after environmental exposure. While overt and covert observations yielded similar overall adherence rates, covert monitoring provided more nuanced insights into true adherence patterns, especially among physicians. These findings highlight the need for tailored educational programs and targeted interventions designed to address the specific needs of different HCW groups, aiming to improve hand antisepsis adherence and ultimately reduce hospital-acquired infections.

According to scientific evidence, hands are the most important factor in transmitting hospital pathogens, and hand antisepsis is the most effective method for prevention and reducing hospital infections [10], [11]. Direct observation of hand antisepsis in healthcare centers is currently considered the gold standard method of monitoring. However, the main limitation of this method is the Hawthorne effect. Although the Hawthorne effect often leads to overestimation of hand antisepsis adherence in hospitals, few studies have precisely evaluated its impact. Covert observation has been used as a tool to quantify or overcome Hawthorne effect bias [4].

The overall adherence rate was 58.4% in overt observations and 60.3% in covert observations with no significant difference (p=0.35). This is in contrast to previous studies that consistently displayed overestimation of hand antisepsis adherence during overt observation, which was usually attributed to the Hawthorne effect [3], [4], [6], [12], [13], [14], [15], [16]. A 2019 meta-analysis by Houghton et al. [17] found that overt observation overestimated hand antisepsis adherence by 15–20%.

According to WHO guidelines and scientific evidence, the recommended duration for effective hand antisepsis with alcohol-based hand rubs (ABHRs) is at least 15 seconds, ensuring complete coverage of all hand surfaces and effective pathogen elimination [6], [18], [19], [20], [21], [22], [23], [24]. Shorter durations reduce microbial removal efficacy, and in high workload settings, inadequate adherence to the recommended duration may contribute to suboptimal hand hygiene [17], [25]. Therefore, training and monitoring should emphasize compliance with the minimum recommended duration, and ABHRs are particularly suitable in ICU settings due to their rapid effect and lower time requirement compared to handwashing with soap and water. Importantly, the findings and recommendations from the 3rd ICPIC ABHR Task Force further support the widespread and consistent use of ABHRs in healthcare settings as one of the most effective measures to prevent hospital-acquired infections. Continuous education, direct monitoring, and adherence to the recommended hand antisepsis duration can significantly improve healthcare workers’ compliance. Combining educational programs with targeted interventions for different healthcare worker groups, especially physicians and nurses, can improve real adherence patterns and reduce the impact of the Hawthorne effect.

Perhaps the difference between this study and previous ones on comparing covert and overt methods lies in the departments evaluated. Our study evaluated adult intensive care units, while previous studies evaluated most ICUs, internal medicine and surgery departments, and emergency departments. Given the sensitive situation of ICUs regarding infection spread and special emergency conditions, hand antisepsis is particularly important, and more education and training is provided about it. ICU senior physicians also more strongly emphasize this topic, and take the lead in hand antisepsis adherence themselves. It seems the study subjects in both observation methods understood the importance and complied with hand antisepsis measures. The covert method showed a small numerical increase in hand antisepsis adherence that may partially be explained by the fact that ICU staff are accustomed to frequent observations, reducing the Hawthorne effect’s impact. Alternatively, the presence of covert observers might have been detectedand increased the adherence, as ICU environments are high-alert zones where unfamiliar personnel could arouse suspicion. 

On the other hand, when groups or individuals realize they are being observed and studied, they may change their behavior. This change can be positive or negative depending on the research context. Therefore, the Hawthorne effect is a subset of bias, and factors like performance feedback (subjects may show improvement or lack thereof based on positive/negative feedback), demand characteristics bias (subjects may feel motivated to please researchers and thus adjust their behavior positively), and novelty effect (when a learning experience is new and implemented for the first time, people perform better, but this effect diminishes over time) can particularly influence the study subject.

Importantly, the similarity in adherence rates implies that both methods are valid for assessing hand antisepsis in this context, although covert monitoring may better capture baseline behavior in less scrutinized areas (e.g., non-ICU wards).

According to this study's findings, there was a significant difference between the two observation methods in terms of physicians' hand antisepsis adherence rates (p<0.0001). In overt observation, nurses, and in covert observation, physicians, had the highest hand antisepsis adherence compared to other staff (62.4% and 66.8% respectively). Previous studies have shown the highest hand antisepsis adherence among nurses compared to physicians and other medical staff [6], [25]. High workload, lack of consequences for non-adherence, low participation in training sessions, and the misconception that wearing gloves eliminates the need for hand antisepsis may be reasons for non-adherence among these groups. The main areas needing improvement for staff acceptance are training and motivation. While training staff about hand antisepsis, emphasis should also be placed on the fundamental principles of handwashing behavior patterns, which can help change individual attitudes.

In the present study, hand antisepsis adherence in surgical ICUs was slightly higher when covertly than overtly observed (p<0.0001). In overt observation, medical ICUs, and in covert observation, surgical ICUs, (61.2% and 69.8%, respectively), had the highest hand antisepsis adherence rates compared to other ICUs. No comparable study matching these findings is available, but previous studies confirm higher hand antisepsis adherence in ICUs compared to other wards [6], [25]. Surgical ICUs admit patients who have undergone major surgeries with subsequent acute dysfunction of one or more vital organs. Given the multiple wounds, frequent daily dressing changes, and longer ICU stays, hand antisepsis is particularly crucial here.

The study found that in both observation methods, ABHRs (87.1%) were preferably implemented compared to handwashing with soap and water (12.9%). Previous studies also confirm this hand hygiene adherence rate [6], [11]. ABHRs perform better than hand washing due to immediate effect, requiring less time, greater pathogen removal efficacy, better skin tolerability, and no need for water [11].

The present study found that both observation methods showed similarities and /or variations in hand antisepsis adherence across different situations. In both methods, the highest adherence rate was after bodily fluid exposure. The lowest adherence in overt observation was after contact with patient surroundings (22%), and in covert observation, the lowest adherence rate was before patient contact (41.5%). Overall, there were significant differences in adherence after contact with patient surroundings (0.46; p<0.0001) and after patient contact (0.89; p=0.039). Previous studies have reported this for before hand antiseptic procedures [6]. Low adherence was found before patient contact (41.5–44.3%), aligning with global data [10]. This suggests HCWs prioritize self-protection (e.g., after exposure to bodily fluids: 84.6–85.5%) over preventing pathogen transmission to patients.

Our observations showed higher acceptance of hand antisepsis when staff perceived personal risk, such as after contact with patient surroundings or patients.

Although this study found no difference in hand antisepsis adherence between the two observation methods, we still believe overt observation is the gold standard for measuring hand antisepsis adherence. It reveals the timing, reasons and technique of hand antisepsis better than does covert observation. Furthermore, this monitoring method is recognized by the WHO and is implemented in various healthcare settings worldwide [1]. Therefore, future research should focus on improving overt methodology to minimize the Hawthorne effect. Furthermore, it is essential to establish hand antisepsis as a culture in healthcare settings. Raising medical teams' awareness about adverse consequences of HAIs can provide the necessary motivation for consistent and proper implementation of this vital practice.

## Notes

### Competing interests

The authors declare that they have no competing interests.

### Ethical approval 

This research was conducted in accordance with the ethical standards of the institutional and/or national research committee and received ethical approval from the Ethics Committee of [Shiraz University of Medical Sciences Ethics Committee], approval code [IR.SUMS.REC.1402.344].

### Funding

None. 

## Erratum

- Change of name of Naeimehossadat Asmarian
- Change of affiliation of Farid Zand and Naeimehossadat Asmarian

## Figures and Tables

**Table 1:**
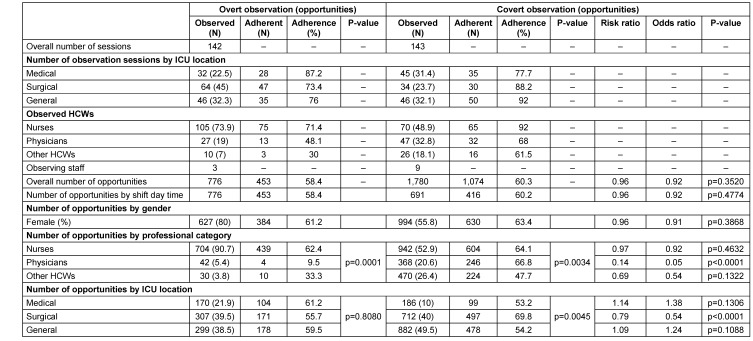
Comparisons of the two observation methods and hand antisepsis adherence by observation method, hospital location

**Table 2:**
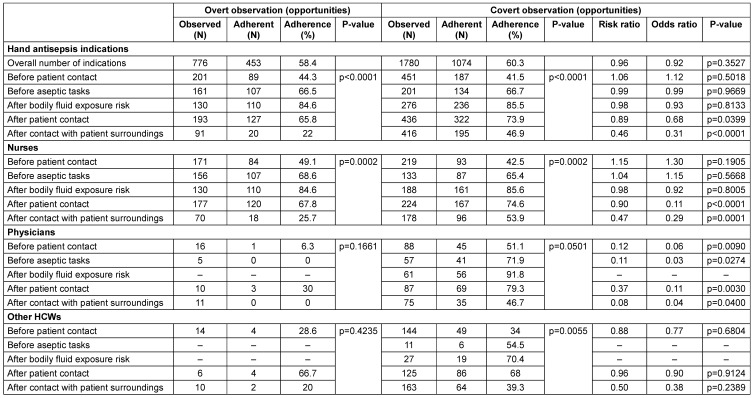
Hand antisepsis adherence by observation method, professional category, and hand hygiene indication

## References

[1] World Health Organization (2009). WHO Guidelines on Hand Hygiene in Health Care: First Global Patient Safety Challenge - Clean Care Is Safer Care.

[2] Pan SC, Tien KL, Hung IC, Lin YJ, Sheng WH, Wang MJ, Chang SC, Kunin CM, Chen YC (2013). Compliance of health care workers with hand hygiene practices: independent advantages of overt and covert observers. PLoS One.

[3] Allegranzi B, Pittet D (2009). Role of hand hygiene in healthcare-associated infection prevention. J Hosp Infect.

[4] Hagel S, Reischke J, Kesselmeier M, Winning J, Gastmeier P, Brunkhorst FM, Scherag A, Pletz MW (2015). Quantifying the Hawthorne Effect in Hand Hygiene Compliance Through Comparing Direct Observation With Automated Hand Hygiene Monitoring. Infect Control Hosp Epidemiol.

[5] Erasmus V, Daha TJ, Brug H, Richardus JH, Behrendt MD, Vos MC, van Beeck EF (2010). Systematic review of studies on compliance with hand hygiene guidelines in hospital care. Infect Control Hosp Epidemiol.

[6] El-Saed A, Noushad S, Tannous E, Abdirizak F, Arabi Y, Al Azzam S, Albanyan E, Al Jahdalil H, Al Sudairy R, Balkhy HH (2018). Quantifying the Hawthorne effect using overt and covert observation of hand hygiene at a tertiary care hospital in Saudi Arabia. Am J Infect Control.

[7] Yoo E, Ursua L, Clark R, Seok J, Jeon J, Kim HB (2019). The effect of incorporating covert observation into established overt observation-based hand hygiene promotion programs. Am J Infect Control.

[8] Bruchez SA, Duarte GC, Sadowski RA, Custódio da Silva Filho A, Fahning WE, Belini Nishiyama SA, Bronharo Tognim MC, Cardoso CL (2020). Assessing the Hawthorne effect on hand hygiene compliance in an intensive care unit. Infect Prev Pract.

[9] Sax H, Allegranzi B, Uçkay I, Larson E, Boyce J, Pittet D (2007). 'My five moments for hand hygiene': a user-centred design approach to understand, train, monitor and report hand hygiene. J Hosp Infect.

[10] Pittet D, Allegranzi B, Boyce J, World Health Organization World Alliance for Patient Safety First Global Patient Safety Challenge Core Group of Experts (2009). The World Health Organization Guidelines on Hand Hygiene in Health Care and their consensus recommendations. Infect Control Hosp Epidemiol.

[11] Kampf G, Kramer A (2004). Epidemiologic background of hand hygiene and evaluation of the most important agents for scrubs and rubs. Clin Microbiol Rev.

[12] Srigley JA, Furness CD, Baker GR, Gardam M (2014). Quantification of the Hawthorne effect in hand hygiene compliance monitoring using an electronic monitoring system: a retrospective cohort study. BMJ Qual Saf.

[13] Wu KS, Chen YS, Lin HS, Hsieh EL, Chen JK, Tsai HC, Chen YH, Lin CY, Hung CT, Sy CL, Tseng YT, Lee SS (2017). A nationwide covert observation study using a novel method for hand hygiene compliance in health care. Am J Infect Control.

[14] Pan SC, Tien KL, Hung IC, Lin YJ, Sheng WH, Wang MJ, Chang SC, Kunin CM, Chen YC (2013). Compliance of health care workers with hand hygiene practices: independent advantages of overt and covert observers. PLoS One.

[15] Kingston L, O'Connell NH, Dunne CP (2016). Hand hygiene-related clinical trials reported since 2010: a systematic review. J Hosp Infect.

[16] Kohli E, Ptak J, Smith R, Taylor E, Talbot EA, Kirkland KB (2009). Variability in the Hawthorne effect with regard to hand hygiene performance in high- and low-performing inpatient care units. Infect Control Hosp Epidemiol.

[17] Houghton C, Meskell P, Delaney H, Smalle M, Glenton C, Booth A, Chan XHS, Devane D, Biesty LM (2020). Barriers and facilitators to healthcare workers' adherence with infection prevention and control (IPC) guidelines for respiratory infectious diseases: a rapid qualitative evidence synthesis. Cochrane Database Syst Rev.

[18] Farhoudi F, Sanaei Dashti A, Hoshangi Davani M, Ghalebi N, Sajadi G, Taghizadeh R (2016). Impact of WHO Hand Hygiene Improvement Program Implementation: A Quasi-Experimental Trial. Biomed Res Int.

[19] Pires D, Soule H, Bellissimo-Rodrigues F, Gayet-Ageron A, Pittet D (2017). Hand Hygiene With Alcohol-Based Hand Rub: How Long Is Long Enough? Infect Control Hosp Epidemiol.

[20] Pires D, Soule H, Bellissimo-Rodrigues F, de Kraker MEA, Pittet D (2019). Antibacterial efficacy of handrubbing for 15 versus 30 seconds: EN 1500-based randomized experimental study with different loads of Staphylococcus aureus and Escherichia coli. Clin Microbiol Infect.

[21] Tartari E, Bellissimo-Rodrigues F, Pires D, Fankhauser C, Lotfinejad N, Saito H, Suchomel M, Kramer A, Allegranzi B, Boyce J, Sax H, Stewardson AJ, Pittet D, ICPIC Alcohol-Based Handrub Task Force (2024). Updates and future directions regarding hand hygiene in the healthcare setting: insights from the 3rd ICPIC alcohol-based handrub (ABHR) task force. Antimicrob Resist Infect Control.

[22] Kramer A, Pittet D, Klasinc R, Krebs S, Koburger T, Fusch C, Assadian O (2017). Shortening the Application Time of Alcohol-Based Hand Rubs to 15 Seconds May Improve the Frequency of Hand Antisepsis Actions in a Neonatal Intensive Care Unit. Infect Control Hosp Epidemiol.

[23] Paula H, Becker R, Assadian O, Heidecke CD, Kramer A (2018). Wettability of hands during 15-second and 30-second handrub time intervals: A prospective, randomized crossover study. Am J Infect Control.

[24] Harnoss JC, Dancer SJ, Kaden CF, Baguhl R, Kohlmann T, Papke R, Zygmunt M, Assadian O, Suchomel M, Pittet D, Kramer A (2020). Hand antisepsis without decreasing efficacy by shortening the rub-in time of alcohol-based handrubs to 15 seconds. J Hosp Infect.

[25] Wu KS, Lee SS, Chen JK, Chen YS, Tsai HC, Chen YJ, Huang YH, Lin HS (2018). Identifying heterogeneity in the Hawthorne effect on hand hygiene observation: a cohort study of overtly and covertly observed results. BMC Infect Dis.

